# Minimizing the source of nociception and its concurrent effect on sensory hypersensitivity: An exploratory study in chronic whiplash patients

**DOI:** 10.1186/1471-2474-11-29

**Published:** 2010-02-09

**Authors:** Geoff M Schneider, Ashley D Smith, Allen Hooper, Paul Stratford, Kathryn J Schneider, Michael D Westaway, Bevan Frizzell, Lee Olson

**Affiliations:** 1 Faculty of Medicine, University of Calgary, Calgary, Alberta, Canada; 2 LifeMark Health, Calgary, Alberta, Canada; 3 Advanced Spinal Care Centre (EFW Radiology), Calgary, Alberta, Canada; 4 Department of Physiotherapy, University of Queensland, Brisbane, Australia; 5 School of Rehabilitation Sciences, Department of Epidemiology and Biostatistics, McMaster University, Hamilton, Ontario, Canada; 6 Faculty of Kinesiology, University of Calgary, Calgary, Alberta, Canada; 7 School of Rehabilitation Sciences, McMaster University, Hamilton, Ontario, Canada; 8 Department of Physical Therapy, Andrews University, Berrien Springs, Michigan, USA

## Abstract

**Background:**

The cervical zygapophyseal joints may be a primary source of pain in up to 60% of individuals with chronic whiplash associated disorders (WAD) and may be a contributing factor for peripheral and centrally mediated pain (sensory hypersensitivity). Sensory hypersensitivity has been associated with a poor prognosis. The purpose of the study was to determine if there is a change in measures indicative of sensory hypersensitivity in patients with chronic WAD grade II following a medial branch block (MBB) procedure in the cervical spine.

**Methods:**

Measures of sensory hypersensitivity were taken via quantitative sensory testing (QST) consisting of pressure pain thresholds (PPT's) and cold pain thresholds (CPT's). In patients with chronic WAD (n = 18), the measures were taken at three sites bilaterally, pre- and post- MBB. Reduced pain thresholds at remote sites have been considered an indicator of central hypersensitivity. A healthy age and gender matched comparison group (n = 18) was measured at baseline. An independent t-test was applied to determine if there were any significant differences between the WAD and normative comparison groups at baseline with respect to cold pain and pressure pain thresholds. A dependent t-test was used to determine whether there were any significant differences between the pre and post intervention cold pain and pressure pain thresholds in the patients with chronic WAD.

**Results:**

At baseline, PPT's were decreased at all three sites in the WAD group (p < 0.001). Cold pain thresholds were increased in the cervical spine in the WAD group (p < 0.001). Post-MBB, the WAD group showed significant increases in PPT's at all sites (p < 0.05), and significant decreases in CPT's at the cervical spine (p < 0.001).

**Conclusions:**

The patients with chronic WAD showed evidence of widespread sensory hypersensitivity to mechanical and thermal stimuli. The WAD group revealed decreased sensory hypersensitivity following a decrease in their primary source of pain stemming from the cervical zygapophyseal joints.

## Background

Cervical spine pain and dysfunction resulting from a motor vehicle collision (MVC) are common patient problems encountered by health care practitioners. Many patients will significantly recover with respect to neck pain and disability within the first six months to one year [1,2]. Researchers have reported that 32% to 56% of those that have sustained a MVC will continue to suffer pain and disability beyond the six month period [3-5].

The cervical zygapophyseal joint has been implicated as a source of pain in those with chronic WAD [6,7]. Studies utilizing controlled, comparative anaesthetic nerve block procedures have reported that the prevalence of cervical zygapophyseal joint pain in those with chronic WAD ranged from 54% to 60% [6,7]. Biomechanical and neurophysiological studies have provided evidence in support of cervical zygapophyseal joint involvement in MVC's [8-29].

Research has indicated that the ongoing pain associated with chronic WAD may be due to altered pain processing as evidenced by sensory hypersensitivity at distant sites involving uninjured tissues [30-34]. Central nervous system hyperexcitability may provide an explanation for the generalized sensory hypersensitivity seen in some patients with chronic WAD [32,33,35]. Sensory hypersensitivity is characterized by decreased pain thresholds to mechanical, thermal, and electrical stimuli [30,31,33,34,36,37]. The presence of sensory hypersensitivity, in particular cold hyperalgesia, in whiplash patients has been associated with a poor prognosis [3,33]. The precise mechanisms underlying sensory hypersensitivity are unclear, but peripheral, spinal, and supraspinal mechanisms have been hypothesized [32,38].

Alterations in neuronal excitability in the spinal cord, secondary to ongoing peripheral nociception, has been hypothesized as a mechanism of central hyperexcitability [39,40]. Contributing to central hyperexcitability is the activation of N-methyl D-aspartate (NMDA) receptors, and subsequent release of cyclooxygenase-2 (COX-2) in the spinal cord, as well as the activation of glial cells [39,41,42]. Clinical manifestations of central hyperexcitability are represented by lowered pain thresholds in areas distant from the site of tissue injury (secondary hyperalgesia) and allodynia. Another contributing factor to central hyperexcitability stems from higher brain centers and is represented by the imbalance in descending facilitatory and inhibitory pathways [35].

Structural injury secondary to trauma may lead to an inflammatory response characterized by the release of inflammatory mediators such as substance P, prostaglandins, and bradykinin [42,43]. As a result of this inflammatory response, peripheral nociceptors may become sensitized. With long periods of nociception, primary hyperalgesia may be maintained as peripheral nerve fibers such as A-fibers, assume C-fiber characteristics [44]. Recently, it has been shown that myofascial trigger points in the upper fibers of the trapezius in subjects with chronic WAD may act as peripheral modulators of sensory hypersensitivity [45]. Measures indicative of mechanical hyperalgesia, taken via pressure pain thresholds over hypothesized injured and uninjured tissues, were increased immediately following local anaesthetic injection of the myofascial trigger points, suggesting an alteration in central pain processing. Contrarily, results of another investigation revealed that anaesthetic injection of painful and tender points in the cervical musculature of chronic WAD subjects did not affect measures indicative of sensory hypersensitivity, leading these researchers to believe that sensory hypersensitivity was not maintained by nociceptive input from these tissues [30]. It is possible that peripherally mediated pain stemming from the underlying cervical zygapophyseal joints may be a source of ongoing nociceptive input into the central nervous system, thus facilitating sensory hypersensitivity.

The aim of this study was to minimize cervical spine pain intensity in patients with chronic WAD and to evaluate its immediate effect on measures indicative of sensory hypersensitivity. We hypothesized that a decrease in cervical spine pain intensity following diagnostic blockade of the cervical zygapophyseal joints would result in a change in measures indicative of sensory hypersensitivity, specifically, an increase in pressure pain thresholds (PPT's) and a decrease in cold pain thresholds (CPT's).

## Methods

### Study Design

This exploratory study involved a pretest-posttest design. A healthy age- and gender-matched normative comparison group was measured at baseline.

### Subjects and Setting

Eighteen volunteers (3 males, 15 females, mean age 45 years ± 8) with whiplash associated disorders grade II as defined by the Quebec Task Force classification (neck complaint and musculoskeletal sign(s) including decreased range of motion and point tenderness) and 18 healthy age- and gender-matched volunteers (3 males, 15 females, mean age 45 years ± 8) participated in this study [46]. Patients with chronic WAD aged 18-60 years, reporting neck pain for greater than 6 months, who experienced at least an 80 percent decrease in familiar neck pain intensity following an intra-articular zygapophyseal joint block procedure were included in the study. From June 2007 to February 2008, the patients with chronic WAD were recruited from a tertiary spinal intervention center in Calgary, Alberta, Canada where they were scheduled for diagnostic cervical zygapophyseal joint blockade. All patients with chronic WAD underwent unilateral cervical medial branch block (MBB) procedures below the C2 level (ie; C3-7) for their predominant neck pain (not headache). The patients with chronic WAD were excluded if they reported a previous history of neck pain or headaches that required treatment. They were also excluded if they were pregnant, had central or peripheral neurological dysfunction, peripheral vascular disease or coronary artery disease. The normative comparison group was recruited through print advertisements at several physiotherapy clinics and medical offices in the surrounding community. The comparison subjects were included if they did not currently report spinal, elbow, knee pain or headache, had not been involved in a MVC, and had not undergone treatment for neck pain or headache in the past 2 years.

Ethics approval was granted by the Centre for Advancement of Health at the University of Calgary (Calgary, Alberta, Canada) and the Institutional Review Board at Andrews University (Berrien Springs, MI, USA).

### Instrumentation

A single item Numeric Pain Rating Scale (0-10) was used to measure the patients' cervical spine pain intensity before and after the MBB procedure. Quantitative sensory testing (QST), consisting of PPT's and CPT's, was performed on the WAD and normative comparison subjects. The CPT's were measured using the TSA II Neurosensory Analyzer (Medoc Advanced Medical Systems; Minneapolis, MN, USA). The PPT's were measured by an electronic pressure algometer (Somedic AB; Farsta, Sweden).

Baseline measures also included a self-report measure of neck pain and disability via the Neck Disability Index (NDI: 0-100) [47]. Demographic variables including gender, age (years), duration of neck pain (months), and litigation status (retained a lawyer) were also recorded.

### Quantitative Sensory Tests

#### Cold Pain Threshold Test

Cold pain thresholds were measured by placing the thermode on the skin over the articular pillars of the cervical zygapophyseal joints that were anaesthetized during the MBB procedure. The thermode size was 30 millimeters × 30 millimeters. The thermode temperature was set to 32°C. The temperature decrease was standardized at a rate of 1°C per second. The minimal temperature was set to zero degrees Celsius. To identify CPT's, the patients were asked to push a self-controlled switch as soon as the cold sensation changed to one of pain. Three tests were performed bilaterally at each site and the mean values were recorded for use in the statistical analyses.

Psychometric properties for cold pain threshold testing are lacking in the literature. In a study in healthy adults, investigators reported good intrarater reliability, ICC's ranging from 0.79 to 0.94, for a clinical test of cold pain threshold [48].

In our study, cold pain threshold testing was only performed at the cervical spine only in order to standardize our testing protocol with other investigations involving subjects with WAD [33,49].

#### Pressure Pain Threshold Test

Pressure pain thresholds were measured at the following sites: the articular pillars of the cervical zygapophyseal joints that were anaesthetized during the MBB procedure, the peripheral nerve trunk of the median nerve (identifiable in the cubital fossa medial to and immediately adjacent to the biceps tendon), and the tibialis anterior (at a site halfway between the most superior attachment to the tibia and its tendon in the upper one third of the muscle belly). The patients were asked to push a self-controlled switch as soon as the sensation of pressure changed to one of pain. The probe size was 1 cm^2 ^and the rate of application was standardized to 40 kPa/sec. Three tests were performed at each site bilaterally and the mean values were recorded for use in the statistical analyses. Ten seconds was allowed between each test.

Pressure pain threshold testing has demonstrated good to excellent intrarater and interrater reliability in patients with chronic WAD, with ICC's ranging from 0.85 to 0.91 and 0.88 to 0.97 respectively [50].

The QST protocols utilized in this study were replicated from previous studies in chronic whiplash patients [33,36,49].

#### Diagnostic Cervical Zygapophyseal Joint Blockade

The patients with chronic WAD were referred to our tertiary spinal care center for diagnostic cervical zygapophyseal joint blockade. This process involved two diagnostic zygapophyseal joint block procedures. Prior to the study, the patients with chronic WAD underwent a diagnostic intra-articular zygapophyseal joint block procedure. For this procedure, a 25-gauge spinal needle is advanced, under flouroscopic guidance, into the target zygapophyseal joint with the patient in the prone position. A small amount of nonionic contrast (0.5 cc of Omnipaque 300^® ^Amerslan Health, Oakville, ON, Canada) was utilized to confirm proper needle position. Subsequently, an injection of 0.5 cc of preservative free 1% Bupivicaine (AstraZeneca, Mississauga, ON, Canada), a local anaesthetic, and 0.5 cc of Celestone (Celestone Soluspan^®^, Schering, Pointe-Claire, Quebec, Canada), a corticosteroid, was made into the target zygapophyseal joint.

The referral source, the general practitioner, physiotherapist, and/or medical specialist determined the spinal level and side of the zygapophyseal joint block based on the patients' clinical presentation. The interventional radiologist confirmed the appropriate target zygapophyseal joint based on clinical examination findings, including established pain diagrams [51,52]. During the post-injection follow-up period, if the patients with chronic WAD reported a decrease in familiar cervical spine pain intensity of at least 80 percent and their pain returned, they underwent a second diagnostic cervical zygapophyseal joint block, the MBB.

The MBB involved the placement of a 25-gauge spinal needle, under fluoroscopic guidance, onto the medial branch of the dorsal ramus as it courses over the waist of the articular pillar at each spinal level. An injection of nonionic contrast material (0.5 cc of Omnipaque 300^® ^Amerslan Health, Oakville, ON, Canada) was made to confirm the proper needle position. Subsequently, 0.5 cc of 2% Lidocaine (AstraZeneca, Mississauga, ON, Canada), a local anaesthetic without preservatives, was injected onto the medial branch of the dorsal ramus. The medial branch of the dorsal ramus provides the sensory innervation to the zygapophyseal joint above and below the target joint as well as the deep paramedian muscles [7,53]. Hence, both medial branches to the target joint need to be anaesthetized in order to effectively anaesthetize one zygapophyseal joint [54].

Controlled comparative MMB procedures have been advocated for the diagnosis of zygapophyseal joint pain [53-55]. In our study, for the diagnosis of zygapophyseal joint mediated pain, we initially performed an intra-articular anaesthetic-corticosteroid facet joint injection to the suspected painful joints. Following this procedure, if the patient experienced at least 80% relief of familiar pain intensity and their pain returned they underwent a diagnostic MBB procedure. Clinically, this diagnostic pathway is used prior to consideration for radiofrequency neurotomy. Although the scientific literature is varied, there is some evidence to suggest that a subset of patients with suspected cervical zygapophyseal joint pain might experience a therapeutic benefit from an intra-articular injection, with respect to a decrease in pain intensity over a period of three months or greater [56]. As our centre provides pain management services to a large catchment area, we possess a four to six month wait-list for interventional techniques including diagnostic zygapophyseal joint procedures. Historically, we utilized a triple-injection procedure for the diagnosis of zygapophyseal joint pain consisting of an intra-articular zygapophyseal joint injection, followed by controlled, comparative MBB procedures. Our unpublished data revealed that it was nearly universal, in that patients that responded positively to both an intra-articular zygapophyseal joint injection and the first MBB, responded positively to the second MBB. By eliminating the second MBB, we were able to reduce patient wait-time by approximately 15-20%.

### Procedures

Following written consent, the patients with chronic WAD reported their pre-MBB cervical spine pain intensity via the NPRS. They also completed the NDI. The whiplash and the comparison groups underwent the QST procedures. The QST were performed in the following order: CPT testing followed by PPT testing. A set order of testing was performed to control for the effect that one test could have on the results of another test [57]. Expected findings were not stated to avoid the potential effect of expectancy bias on the test results [57]. All tests were performed by one of the investigators (GS). The instructions provided to the subjects were standardized. The test sites were measured in a random fashion in order to minimize a learning effect. The comparison group underwent QST testing at baseline only to allow for a statistical comparison of differences in PPT's and CPT's between the this group and the patients with chronic WAD.

Within 30 minutes of the baseline QST protocol, the patients with chronic WAD underwent their scheduled MBB procedure. If the patient reported an 80 percent or greater relief in cervical pain intensity (via the NPRS) within one hour post-MBB, they underwent a final round of QST [58]. As the MBB procedure involves a needle puncture, total pain relief is not always a realistic outcome secondary to procedural discomfort. If the patients with chronic WAD did not report at least an 80 percent decrease in cervical spine pain intensity they did not continue in the study.

### Data Analysis

Descriptive and inferential statistics were applied to describe the baseline characteristics of the WAD and normative comparison groups. Sample means and standard deviations were applied to continuous data. Frequency counts were used to summarize categorical data. Reliability analyses, ICC (2,3), along with standard error of measurement calculations were performed on the left and right side mean PPT and CPT data at the three sites on the WAD group pre-and-post-MBB and the normative comparison group at baseline. If the absolute agreement was acceptable, then the mean left and right side data were averaged and a point estimate representative of each body region were used for further analyses.

An independent t-test was applied to determine if there were any significant differences between the WAD and normative comparison groups at baseline, and following the MBB, with respect to cold pain and pressure pain thresholds. A dependent t-test was used to determine whether there were any significant differences between the pre and post-MBB cold pain and pressure pain thresholds in the patients with chronic WAD. The statistical software used to analyze the data was STATA 10.1 (StataCorp, College Station, TX, USA). Significance level was set at p < 0.05.

### Sample Size

An a priori sample size analysis indicated the need for at least 18 subjects in each group. For the sample size analysis, considering the requirements for a paired t-test, the investigators incorporated the following parameters: power of 0.80, effect size estimate of 0.60 (medium effect size)[59], probability of making a type II error (beta) of 0.20, probability of making a type I error (alpha) of 0.05. As previous research in this area was not available at the time of our study, a medium effect size was chosen based on our consensus of clinically meaningful differences, considering the inclusion criteria of an 80% decrease in self-reported pain intensity post-MBB [60]. This study incorporated a directional, one-tailed hypothesis.

## Results

### Group Characteristics

Table 1 presents the baseline characteristics for the WAD and normative comparison groups. The two groups did not differ significantly with respect to age (p = 0.94) and gender (15 females and 3 males in each group). Fifty percent of the patients with chronic WAD were involved in litigation for compensation for their injuries at the time of the study. Eighteen subjects that initially underwent diagnostic intra-articular facet joint injections went on to receive the diagnostic MBB procedure. Subsequently, all 18 subjects were examined at all measurement time-points in the study as all of them experienced at least 80 percent relief in familiar neck pain intensity following the MBB. Thus, there were no exclusions from the post-MBB QST testing.

**Table 1:** Baseline Group Characteristics

*Group* *(n)*	*Gender* *(% M/F)*	*Age* *(yrs ± SDŧ)*	*NPRS§* *(± SDŧ)*	NDI* (± SDŧ)	*Duration of symptoms (mos ± SDŧ)*
WAD (18)	83/17	45 (8)	6 (1)	44 (13)	27 (16)

Comparison (18)	83/17	45 (8)	--	--	--

Groups did not differ in age (p = 0.94) and gender

§ NPRS - Numeric Pain Rating Scale

* NDI - Neck Disability Index

∓ SD - Standard deviation

The reliability analyses performed on all pairs of body parts for the CPT's and PPT's, pre-and post-MBB, in the WAD and normative comparison groups revealed ICC's (2,3) ranging from 0.977 to 0.994 (95% CI: 0.955 - 0.997) [61]. The standard error of measurement (SEM) associated with the QST protocol ranged from 0.49 to 0.60 for CPT and 8 to 20 for PPT. Considering the excellent agreement between left and right side CPT and PPT data, the mean left and right side data were averaged and a point estimate representative of each body region was used for further analyses [61].

### WAD versus Normative Comparison Group at Baseline

The mean CPT and PPT data, for the cervical spine, median nerve, and tibialis anterior test sites in the normative comparison and WAD groups, are summarized in Table 2.

**Table 2:** Mean (95% CI) CPT's and PPT's for WAD and Normative Comparison Groups

	WAD pre-MBB	WAD post-MBB	Control
**CPT Cervical** **(95% CI)**	9.6 (5.7-13.5)	3.5 (1.2-5.9)	0.12 (0-0.3)
**PPT Cervical** **(95% CI)**	165 (124-206)	232 (184-281)	348 (301-395)
**PPT Median Nerve** **(95% CI)**	217 (174-258)	245 (195-295)	371 (343-400)
**PPT Tib Ant** **(95% CI)**	350 (288-412)	381 (310-452)	569 (528-608)

CPT = Cold Pain Threshold (degrees Celsius)

PPT = Pressure Pain Threshold (kPa)

Tib Ant = Tibialis Anterior

#### Cold Pain Thresholds

**Figure 1:**
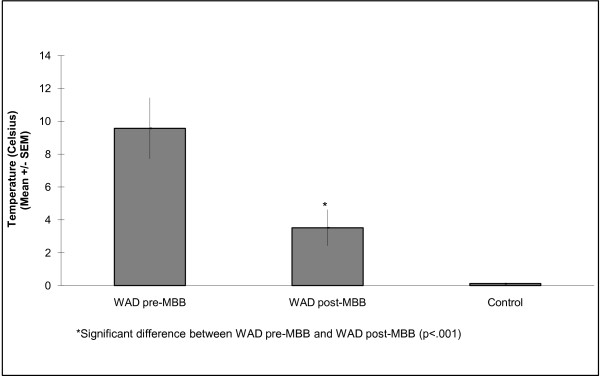
**Mean (standard error) cold pain thresholds in the WAD and normative comparison groups**.

An independent samples t-test revealed significant differences in CPT's at the cervical spine between the WAD and normative comparison groups (p < 0.001) at baseline, with the WAD group having significantly reduced CPT's (Fig. 1).

#### Pressure Pain Thresholds

**Figure 2:**
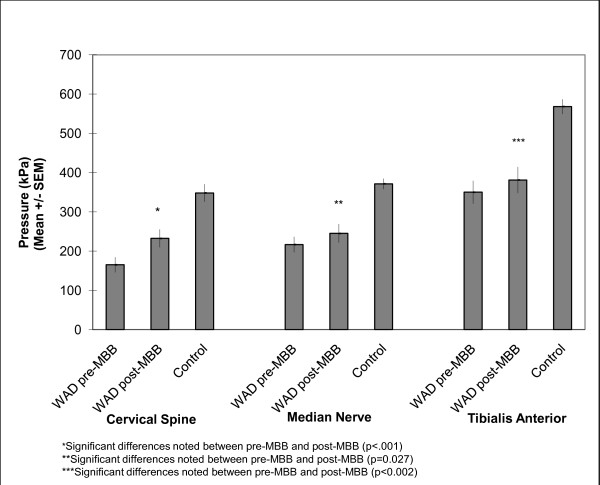
**Mean (standard error) pressure pain thresholds in the WAD and normative comparison groups**. The abbreviations for the figures are as follows: MBB - medial branch block, WAD - whiplash associated disorder, SEM - standard error of the mean.

An independent samples t-test revealed significant differences in PPT's at all sites between the WAD and normative comparison groups (p < 0.001) at baseline, with the WAD group having significantly lower PPT's (Fig. 2).

### WAD pre-MBB versus WAD post-MBB

#### Cold Pain Thresholds

A paired samples t-test revealed significant differences in cervical spine CPT's in the WAD group from baseline to post-MBB (p < 0.001), with this group demonstrating a significant decrease in CPT's post-MBB (Fig. 1).

#### Pressure Pain Thresholds

A paired samples t-test revealed significant differences in the PPT's in the WAD group at all sites from baseline to post-MBB (p < 0.05), with this group demonstrating significant increases in PPT's post-MBB (Fig. 2).

### WAD post-MBB versus Normative Comparison Group

#### Cold Pain Thresholds

An independent samples t-test revealed significant differences in CPT's at the cervical spine between the WAD post-MBB and normative comparison groups (p = 0.004), with the WAD post-MBB group having significantly reduced CPT's.

#### Pressure Pain Thresholds

An independent samples t-test revealed significant differences in PPT's at all sites between the WAD post-MBB and normative comparison groups (p < 0.001), with the WAD post-MBB group having significantly lower PPT's.

## Discussion

The patients with chronic WAD in this study demonstrated evidence of sensory hypersensitivity reflected by reduced pain thresholds to cold temperature and pressure stimulation in the cervical spine as well as reduced pressure pain thresholds at distant sites over the peripheral trunk of the median nerve at the elbow and over the tibialis anterior. These findings are similar to those found in other studies investigating sensory hypersensitivity in patients with chronic WAD [33,57,62]. Evidence of altered pain thresholds to stimuli at distant regions away from the region of reported pain symptoms has been attributed to altered central pain processing [30,31,33,34,63].

Following anaesthetic blockade of the cervical zygapophyseal joint, and a subsequent significant decrease in self-reported cervical spine pain intensity, the patients with chronic WAD demonstrated significant changes in measures indicative of sensory hypersensitivity. Specifically, the WAD group revealed a decrease in CPT's and an increase in PPT's in the cervical spine and, importantly, an increase in PPT's at the distal sites examined. To our knowledge, this is the first study to reveal such findings in patients with chronic WAD. These findings suggest that sensory hypersensitivity may be modulated by minimizing a potential peripheral source of pain, the zygapophyseal joint, in patients with chronic WAD. The prompt increase in local PPT's and decrease in unilateral CPT's (measured over the anaesthetized zygapophyseal joint) noted in the WAD group post-MBB may have been the result of reduced peripheral sensitization via a reduction in receptive fields and/or deactivation of glial cells, thus mitigating primary hyperalgesia in the injured area [32,64,65]. Subsequently, the prompt increase in distal PPT's and decrease in bilateral CPT's in the WAD group post-MBB reflects alterations to central nervous system hyperexcitability. Although not thought of to respond rapidly, the reduction in central hyperexcitability may have been due to inhibition of COX-2 within the central nervous system [42,66]. Animal experimentation has provided evidence for the mediatory effects of COX-2 inhibition on excitatory mechanisms within the central nervous system [67].

Recently, it has been shown that myofascial trigger points in the upper fibres trapezius of patients with chronic WAD may be a peripheral source modulating central hyperexcitability as evidenced by a decrease in widespread mechanical hyperalgesia (pressure pain thresholds) following local anaesthetic injections [45]. Of interest, cold hyperalgesia was not measured in this study. As the presence of sensory hypersensitivity, predominantly cold hyperalgesia, has been associated with a poor prognosis in patients with chronic WAD, it would have been valuable to see the modulatory effects of the myofascial trigger points on both widespread mechanical hyperalgesia and cold hyperalgesia [3,68]. On the contrary, previous research failed to show changes in measures indicative of central hyperexcitability following injection of local anaesthesia into painful and tender points in patients with WAD [30]. The authors concluded other anatomical sources in the cervical spine might have upheld central hyperexcitability. Central hyperexcitability may provide an explanation for the generalized sensory hypersensitivity seen in some patients with chronic WAD [32,33,35]. In our study, it is plausible that the significant decrease in pain intensity reported by the patients with chronic WAD post-MBB resulted in decreased nociceptive input into the central nervous system with subsequent decreased excitability of central nervous system neurons and/or facilitation of inhibitory pathways [35,38].

There are limitations of our study that warrant further discussion. Although the WAD group demonstrated significant changes in the measures of sensory hypersensitivity in the post-MBB follow-up period, conclusions cannot be made about long-term outcomes based on the study design. Without comparison to a placebo group, one cannot conclude that the outcomes of our study were specific to the facet joint block procedure or as a result of repeated testing. Although, our study hypothesis focused on outcomes related to measures of sensory hypersensitivity following a marked decrease in cervical spine pain intensity, a potential peripheral pain mechanism, not the efficacy of the facet joint block procedure itself.

Based on the typical duration of the anaesthetic, 2% Lidocaine (2 hours), used for the MBB procedure in our study, we assume that the measures of sensory hypersensitivity eventually returned to baseline in the subjects with chronic WAD. We cannot discuss this with absolute certainty as the post-MBB measurement period was complete within one hour of the MBB procedure and no further follow-up was performed. Post-MBB, though the WAD group improved with respect to measures of sensory hypersensitivity, the WAD group remained statistically different from the normative comparison group. This suggests that the WAD group was still sensitized, to a certain extent, with respect to pressure pain and cold pain thresholds, in comparison to healthy individuals. Our results propose that minimizing pain intensity from a peripheral source of nociception may modulate sensory hypersensitivity, but may not amend central hyperexcitability to normative levels. This is most likely the result of the complex interaction between peripheral, spinal, and supraspinal mechanisms involved in central hyperexcitability [32,39,69].

In consideration of the diagnostic zygapophyseal joint blockade procedures used in our study one cannot rule out a false positive response with absolute certainty. Comparative local anaesthetic blocks have been advocated to guard against such responses [54]. This involves the administration of two different local anaesthetics on different occasions. The patient should, ideally, note pain relief that coincides with the duration of the anaesthetic used. In our study, as described in the methods section, two diagnostic injection procedures were used. The first diagnostic procedure involved an intra-articular zygapophyseal joint injection while the second diagnostic procedure involved a medial branch block. This combination of diagnostic techniques possesses a similar construct to comparative MBB's and, to our knowledge, has not been refuted in the scientific literature. For both of our diagnostic procedures, target specificity was determined by the use of a contrast medium to ensure needle location [55]. In the case of the intra-articular zygapophyseal joint injections, there were no incidences of a radiate spread of contrast medium; indicative of a lack of target specificity with failure to isolate the zygapophyseal joint [55]. Furthermore, all patients in our study reported an 80% or greater decrease in pain intensity after both diagnostic injection procedures without prior knowledge of expected outcomes leading us to believe that they were legitimate in their responses. The patients were not aware of our hypothesis with respect to changes in measures indicative of sensory hypersensitivity. As the outcomes of our study revealed changes in sensory hypersensitivity following a significant decrease in self-reported pain intensity in the cervical spine, one may speculate that our diagnostic injection procedures targeted a primary source of pain.

Although our study incorporated standardized measures of sensory hypersensitivity that have been detailed in published research [33,36,49], they are subjective in nature. The use of objective measures of sensory hypersensitivity, such as the nociceptive withdrawal reflex [31], and the inclusion of psychological factors in the analysis of outcomes may provide more inclusive evidence in research involving patients with chronicWAD [70,71]. In our study, one examiner that was not blinded to the study hypothesis performed the QST procedures. Although observation bias [60] may be introduced with such procedures, the QST protocol was standardized and the individuals being examined had control of when to cease each trial, thus minimizing the potential of bias in our evaluation.

Based on the exploratory nature of our study, a cause and effect relationship cannot be ascertained, but our data illustrates that sensory hypersensitivity may be modulated in the short-term. As the study sample included a specific subset of the chronic WAD population, the results of the study may not necessarily be generalizable to all patients with chronic WAD. Future studies including the measurement of cold pain thresholds at distal sites may provide additional valuable information in the context of underlying pain mechanisms in patients with chronic WAD. Further research, incorporating longitudinal designs, examining the effects of interventions aimed at minimizing the primary source of cervical spine pain in patients with chronic WAD on measures of sensory hypersensitivity, as well as other clinical and functional outcome measures, is warranted.

## Conclusion

Our exploratory trial reveals a change in measures indicative of sensory hypersensitivity in patients with chronic WAD following a medial branch block procedure in the cervical spine. This suggests that sensory hypersensitivity may be modulated in the short-term when a primary source of pain is reduced. Large clinical trials involving long-term follow-up of interventions aimed at reducing or eliminating the primary source of cervical spine pain in patients with chronic WAD are necessary.

## Competing interests

The authors declare that they have no competing interests.

## Authors' contributions

GS: research design, data collection and statistical analysis, manuscript preparation and revision. AS: research design, manuscript preparation and revision. AH: research design, manuscript preparation and revision. PS: research design, statistical analysis, manuscript preparation and revision. KS: research design, data analysis, manuscript preparation and revision. MW: research design, manuscript preparation and revision. BF: research design, manuscript preparation and revision. LO: research design, data analysis, manuscript preparation and revision. All authors read and approved the final manuscript.

## Pre-publication history

The pre-publication history for this paper can be accessed here:

http://www.biomedcentral.com/1471-2474/11/29/prepub
